# Targeted Myocardial Restoration with Injectable Hydrogels—In Search of The Holy Grail in Regenerating Damaged Heart Tissue

**DOI:** 10.3390/biomedicines9060595

**Published:** 2021-05-24

**Authors:** Faizus Sazzad, Michał Kuzemczak, Engracia Loh, Wellington Wu, Theo Kofidis

**Affiliations:** 1 Myocardial Experimental Laboratory, Department of Surgery, Yong Loo Lin School of Medicine, National University of Singapore, Singapore 117599, Singapore; surmfs@nus.edu.sg (F.S.); engracia_loh@u.nus.edu (E.L.); wellingwu118@gmail.com (W.W.); 2 Department of Invasive Cardiology, Central Clinical Hospital of the Ministry of Interior and Administration, 02-507 Warsaw, Poland; michal.kuzemczak@gmail.com; 3 Department of Medical Rescue, Poznan University of Medical Sciences, 60-806 Poznań, Poland; 4 Department of Cardiac, Thoracic and Vascular Surgery, National University Health System, Singapore 119228, Singapore

**Keywords:** hydrogel, extracellular matrix hydrogels, myocardial infarctions, myocardial infarction therapy, cardiac stem cell therapy, tissue engineering, cell-based therapy

## Abstract

A 3-dimensional, robust, and sustained myocardial restoration by means of tissue engineering remains an experimental approach. Prolific protocols have been developed and tested in small and large animals, but, as clinical cardiac surgeons, we have not arrived at the privilege of utilizing any of them in our clinical practice. The question arises as to why this is. The heart is a unique organ, anatomically and functionally. It is not an easy target to replicate with current techniques, or even to support in its viability and function. Currently, available therapies fail to reverse the loss of functional cardiac tissue, the fundamental pathology remains unaddressed, and heart transplantation is an ultima ratio treatment option. Owing to the equivocal results of cell-based therapies, several strategies have been pursued to overcome the limitations of the current treatment options. Preclinical data, as well as first-in-human studies, conducted to-date have provided important insights into the understanding of injection-based approaches for myocardial restoration. In light of the available data, injectable biomaterials suitable for transcatheter delivery appear to have the highest translational potential. This article presents a current state-of-the-literature review in the field of hydrogel-based myocardial restoration therapy.

## 1. Introduction

### 1.1. “Stem Cells Are the Future of Heart Treatment, and They Will Always Be” Norman Shumway

This may constitute a somewhat nihilistic approach, from the mouth of an authority in cardiac surgery and heart failure treatment, yet holds more or less true to this day—simply taken from the perspective of clinical implementation—in the form of a comprehensive, recommended, if not guideline-supported, protocol: after 25 years into myocardial restoration attempts following myocardial injury, there has not been a single efficient, robust, and sustained impact on the injured heart muscle following ischemic insult. Approaches so far have encompassed various types of cells, cell products or derivatives, scaffolds of various physical conditions, as well as multiple administration routes. It would be beyond the scope of the present paper to revisit them all; however, in brief, they all hold promise and peril.

### 1.2. The Unique and Complex Structure of a Healthy and Injured Myocardium

**Figure 1:**
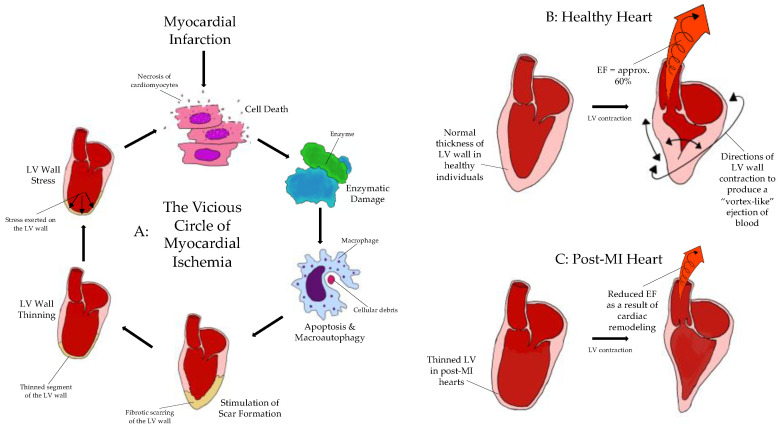
Vicious circle of myocardial ischemia and ventricular wall remodeling after MI. EF: ejection fraction. (**A**): representative diagram of the vicious circle of myocardial ischemia, kickstarted by an initial myocardial infarction. (**B**): contraction of the LV in a healthy heart and the EF produced as a result of efficient contraction. (**C**): contraction of a post-MI heart and the reduced EF produced as a result of altered cardiac architecture.

Gerard Buckberg, with his seminal paper “The Helix and the Heart”, has awakened many aspired myocardial restorers to the fact that the heart is not a quiescent, homogenously built target, but rather is a highly asymmetric, anisotropic, and angiotropic organ, featuring an intricate architecture [1]. Not one spot in the heart is built like another. The heart muscle is not one continuous layer, but rather three layers superimposed at any given point, which can be folded and unfolded like a ribbon, as demonstrated by Buckberg [1]. This leads to a systematic overlay of the three layers at any given spot and the formation of critical intercalations and physical shear stresses, which are organized in an optimal fashion, to: form an oval-shaped vortex and maximize contractile force at the best energy economy. This explains why a systolic diameter increase of only around 8% at the myofiber level translates into a disproportionately higher fractional shortening, ejection fraction, and left ventricular wall thickening [2] (Figure 1B). The arrangement of myofibers, their communications and intercalations, the electrical signal propagation, the fibrous skeleton, and the arrangement of supplying arterioles, all render the myocardium a very anisotropic target, where the random injection of cells of any kind remains rather unimpressive, in terms of real health gain and symptomatic relief, from a clinician’s point of view.

### 1.3. The Vicious Circle of Myocardial Ischemia and the Mechanics of Remodeling

Vu et al. had postulated that the acute myocardial injury, known as infarct, triggers a cascade of events with severe cellular and functional impacts [2]. This vicious circle is self-perpetuating, resulting in the so-called “non-ischemic expansion of the infarct”, unrelated to and not dependent on further coronary occlusions (Figure 1A). This is largely due to a mechanical shift of the myocardial plates and a series of biological phenomena with architectural sequelae. When acute myocardial ischemia and injury manifest, cell death ensues. Enzymatic damage to the tissue is next, with the release of so-called “danger signals” (derivatives of purine metabolism, free radicals, etc.), causing macrophagy and apoptosis [3]. This perpetuates the cell death cycle, stimulating remodeling mechanisms that result in scar formation. As a result, the affected myocardium thins out, while the surrounding myocardium may become temporarily dysfunctional as well. When the LV wall thins out, the modified Laplace law [4] of the oval of the heart takes effect, thus leading to extreme circumferential wall stress, more cell death [5] and architectural remodeling [2], and a drop of contractility and ejection fraction [1,2] (Figure 1C), as compared to that in the heart of a healthy individual [6] (Figure 1B). The outcome is proportional to the extent of tissue loss and dysfunction and may encompass multiple segments of the LV; this is best captured by nuclear scans and MRIs.

### 1.4. Cell-Based Therapy: Unfulfilled Hopes or Misguided Expectations? Why Not Only Cells?

The prevailing dogma suggesting that adult mammalian cardiomyocytes are post-mitotic cells with no ability to renew has been recently overthrown by studies demonstrating a low level of proliferation, even in adult hearts [7]. However, the regenerative capacity is minimal and insufficient to overcome the loss of cardiac cells following MI. The inability of the adult heart to regenerate has yielded several preclinical and clinical studies focused on different cell-based therapies. Despite very promising preclinical results, these results have so far not been translated into clinical practice. Some of the major challenges limiting their clinical application are low retention and survival rates; very limited trans-differentiation into cardiomyocytes; safety; and, in some cases, ethical concerns.

**Figure 2:**
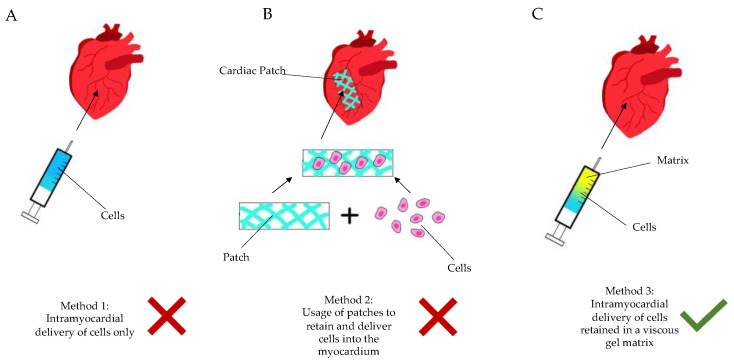
Diagram showing the current methods of regenerative cardiac therapy. (**A**): Method 1—intramyocardial delivery (injection) of stem cells only, without a retaining matrix. (**B**): Method 2—seeding of cells into a patch-like matrix, which is then implanted onto the epicardium via sutures or glue. (**C**): Method 3—intramyocardial injection of cells/active ingredient retained in a gel matrix, either during an open surgery (thoracotomy) or in a minimally invasive manner (percutaneously, etc.).

Over the last few decades, cell therapy has been applied in clinical myocardial restoration. Though the result is non-conclusive, some studies have shown the attenuation of ventricular remodeling. The ensuing hostile and inflammatory environment results in the rapid death of injected cells, or lack of integration thereof. It is incomprehensive, and the vast majority of studies have proven that injected cells do not organize in an integrated syncytium, which excites orchestrated contractility. Depending on the type of cells that are randomly injected, different complications occur [8]. Solid scaffolds—even when adding thickness to the aneurysmatic scar—have not proven themselves as a viable solution either, particularly due to the necessity of open heart surgery to implant them (Figure 2).

There is an obvious need for a targeted, less invasive myocardial restoration treatment, which does not add too much stand-alone trauma to the patient and can be integrated into a viable clinical protocol, to be adopted by cardiologists as well. Arising from the above pain-points, we have long shifted our focus from stem cells to liquid compounds, with the following key value propositions:

1. Injectable, hence minimally invasive, administration
2. Autologous material, not of stem cell nature, to be derived simply during treatment
3. A polytherapy approach to address concomitant aspects of the vicious circle of myocardial ischemia (antioxidants, purine metabolism blockers/anti-inflammatory drugs)
4. Easy adoption and clinical penetration in the horizon.

## 2. Materials and Methods

A literature search was performed electronically using the Preferred Reporting Items for Systematic Reviews and MetaAnalyses (PRISMA) guidelines [9]. We conducted record scrutiny on Medline (via PubMed), Embase, and Web of Science from inception to 31st March 2021. A repetitive and exhaustive combination of the following ‘medical subject headings’ (MeSH) terms were used: “hydrogels“, “extracellular matrix hydrogels“, “tissue engineering“, “myocardial infarctions“, “myocardial infarction therapy“, “cardiac stem cell therapy“, and “f‘cell-based therapy“. This study protocol was registered with PROSPERO #CRD42021250140. The full search strategy can be found in the supplementary materials (Supplementary Figure S1). Relevant articles were screened and systematically assessed with inclusion and exclusion criteria applied.

The inclusion criteria included any experimental cohort studies in which large animals or patients underwent an injectable delivery of hydrogel and/or hydrogel compound analogure for an effect analysis on ischemic heart disease. Furthermore, only studies published after the year 2000 were included to prevent using outdated data. Articles with hydrogel compound processing (lab experiment) and in-vitro experiments, small animal studies, and case reports were excluded. Additionally, any studies that were not written in the English language were excluded. Three authors (E.L., W.W., and F.S.) independently abstracted details of the study characteristics, the myocardial infarct (MI) creation, the hydrogel characteristics, the delivery method, and the outcomes measured. Data extracted, with respect to the infarct creation, hydrogel characteristics, and the delivery method, included: method of MI creation, the artery involved, the cell delivered via hydrogel or its analogues, the type of matrix, and the method of delivery to the myocardium. Data extracted, with respect to the outcomes measured, included: any data related to the functional and morphological outcomes of the heart. Outcomes were then grouped according to the modality they were measured with. All outcomes are expressed as the treatment group outcome when compared to the control group.

## 3. Findings

The systematic search revealed a total of 28,704 papers. After 13,775 duplicates were excluded, 14,929 papers remained for screening. Based on the title and abstract, irrelevant articles were excluded, leaving 70 papers for full-text review. Out of these 70 papers, 69 could be retrieved. Following a full-text review of these papers, 19 papers remained for inclusion. Additional sources provided 2 papers that were added to the final pool, resulting in a total of 21 papers for inclusion into the present study (Supplementary Figure S2). The characteristics of the study population are summarised in Table 1. The experimental groupings and aims of the included studies, including 613 large animals and 15 human subjects, have been plotted. The study characteristics did not differ markedly in their aim, but there was diversity observed in the groupings used and in the use of animal subjects. In some studies, including Zhou et al. [10] and Liu et al. [11], the recipients’ age and sex were not categorized. The hydrogel characterization and its mode of delivery are tabulated in Table 2. Few studies reported the delivery of injectable hydrogel without a cellular component [12,13,14,15,16,17,18], while the rest chose a different composition of cells and type of matrix.

### 3.1. Treatment with Hydrogel Improves Systolic and Diastolic Cardiac Function

Out of the 21 studies, 17 measured systolic function via LVEF and 4 via SV (Table 2). Yamamoto et al., 2001 [21], Chang et al. [25,28], and Li et al. [27] also measured diastolic function via LV EDP. All of the studies that measured LVEF reported an increase, with the exception of the Yamamoto et al. [21] study, which reported an equivocal outcome. These parameters were frequently measured via three main modalities: echocardiography, magnetic resonance imaging (MRI), or ventricular catheterization. Out of the 21 studies, only Leor et al. [12] did not measure functional outcomes of the treatment group.

### 3.2. Treatment with Hydrogel Attenuated LV Remodeling

The ventricle size was frequently measured via ESV, EDV, wall thickness, and LV mass. The common modalities used to measure these parameters included: echocardiography, MRI, and immunohistochemistry. Out of the 21 studies, Giordano et al., 2013 [19], Yamamoto et al., 2001 [20], Zhou et al., 2012 [10], Liu et al., 2006 [11], and Takehara et al., 2008 [29] did not measure the direct effect of treatment on LV remodeling. The remaining 16 studies that looked into LV remodeling reported either attenuated or equivocal LV remodeling. This would suggest that injectable hydrogels have the ability to retain the highly complex and intricate architecture of the heart post-MI, resulting in increased EF [2]. This would be in line with the findings of studies [13,14,15,16,17,18,20,22,23,24,25,26,27,28,30], which reported both attenuated LV remodeling and an increase in EF.

### 3.3. Treatment with Hydrogel Reduces Cardiac Fibrosis

Cardiac fibrosis was frequently quantified via scar size and the extent of fibrosis. These parameters were measured via immunohistochemistry, Masson’s trichrome stain, MRI, and computed tomography (CT). Out of the 21 studies, only 5 studies [10,13,14,23,28] measured the effect of treatment on fibrosis, with Zhou et al. [10] reporting equivocal scar size and the rest reporting reduced fibrosis with treatment.

### 3.4. Treatment with Hydrogel Supports Angiogenesis Post-Infarction 

The degree of angiogenesis was mainly quantified using blood vessel density via immunohistochemistry staining. Out of the 21 studies, only 11 [10,11,14,19,21,23,25,26,27,28,30] measured blood vessel density; each of the 11 studies reported an improved effect, implying that hydrogel treatment can have a positive effect on angiogenesis post-MI. Zhou et al. [10] particularly focuses on the density of specific blood vessels—namely arterioles, small vessels, and larger arterioles—all of which show an increase in density.

## 4. Discussion

### 4.1. Post-MI Survivability

In the vast majority of cases, MI is a consequence of a vulnerable plaque rupture and a subsequent intracoronary thrombosis. The process initiates maladaptive changes in the myocardium, termed “cardiac remodeling“, which may result in the development of HF (Figure 2). The clinical sequelae are encountered in up to three-quarters of patients within five years after their first coronary event [31]. Importantly, HF has not only a significant impact on patients‘ functional capacity and quality of life, but the disease also significantly affects their life expectancy. Available data indicate that approximately half of the patients with HF do not survive for more than five years after the diagnosis [32], meaning that despite the advances in cardiac care, survival rates in this patient population are still very poor and are comparable to those observed in many types of cancer [33,34]. Given the above, more still needs to be done to tackle the burden of the disease more efficiently, thus triggering alternative mono- or poly-therapeutic treatments using viable matter and scaffolds.

### 4.2. Injectable Hydrogel-Based Approach for Cardiac Tissue Engineering

Owing to the intricate myocardial architecture and function, we believe that the triple approach (i.e., enhancing viability, counteracting inflammation, and stabilizing the diminishing architectural integrity of the left ventricle) yields the best restorative effect. Some of the most promising therapeutic compounds are hydrogel-based biomaterials that can not only provide mechanical support for a failing heart, but can also serve as a vehicle for cells, growth factors, and drugs. Because of their potential for minimally invasive transcatheter delivery, injectable hydrogels appear to be one of the most promising types of compounds in terms of their potential clinical application. Several types of hydrogel-based approaches for cardiac tissue repair have been investigated to date. Each category of hydrogels has its advantages and disadvantages that can influence their potential clinical applicability. There are various types of hydrogels with different properties based on their origin (natural/synthetic), various mechanisms of cross-linking, etc.

Based on the best evidence, we have observed a diverse range of compounds with none of the compositions showing clear superiority (Table 3). There are a number of studies, using acellular hydrogel by changing the matrix composition, more focused to investigate whether hydrogel characteristics (i.e, stiffening) enhances therapeutic efficacy to limit LV remodeling and heart failure [16]. Synthetic hydrogel: poly (NIPAAm-co-HEMA-co-MAPLA) (Sigma-Aldrich, St. Louis, MO, USA) was used in some studies [13,17], but hyaluronic acid-based hydrogel was used in most cases. Cell types, including skeletal myoblasts (SKMs), CMs, and other progenitor cells capable of differentiation to CMs, like embryonic stem cells (ESCs), ESC-derived CMs (ESCCMs), and mesenchymal stem cells (MSCs) with limited potentials, were investigated. Human umbilical mesenchymal stem cells [hUMSC] are a new focus [20], whereas basic fibroblast growth factor (bFGF), acidic gelatin hydrogel microspheres (AGHM), and vascular endothelial growth factor (VEGF) are in use with non-superiority to each other. Vu et al. used hyaluronic acid-based hydrogel coupled with PRP and showed improved host-cell viability [30]; while Traverse et al. [18] reported the first-in-human study with VentriGel^TM^ (ECM from the decellularized porcine myocardium) in patients with 1st STEMI treated by PCI within a period of post-intervention between 60 days and 3 years, and found MRI evidence of LV remodeling and a clinical improvement in the study subgroup. 

### 4.3. Less Invasive Administration Modes 

**Figure 3:**
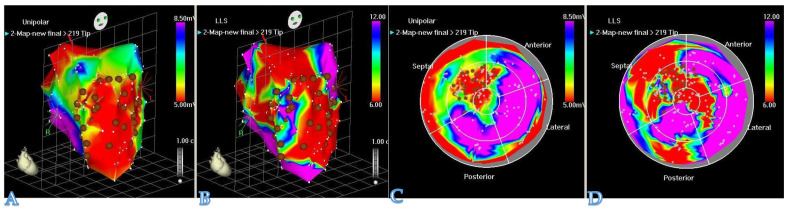
**The** NOGA^®^ system allows visualization of the LV contraction in 3D. NOGA is able to map the heart in a full 360° rotation. (**A**,**C**): unipolar voltage maps (mV) and (**B,D**): regional wall motion maps by local linear shortening (LLS%) can help the electromechanical assessment of the myocardium. NOGA shows viability on the left column; dense scarring is visible at the apex and the antero-septal wall (red); scar area (<0.5 mV) = RED; viable tissue (>1.5 mV) = PURPLE. Comparing the bipolar and unipolar maps, NOGA is able to define border zone areas better.

One of the most important aspects of hydrogel-based myocardial restoration therapy is the mode of delivery. In the context of the increasing role of minimally invasive techniques, a particular emphasis has been placed on shifting away from open heart surgery to catheter-based techniques. We doubt that any restoration method involving major surgical trauma can survive as a stand-alone treatment, as no patient, cardiologist, or surgeon will adopt it. Second, therapy may have to be chronic and repeated (i.e., multiple sessions during the process of time post-MI as HF chronifies). The patient cannot undergo countless re-dos if the procedure is invasive. This has prompted researchers to develop new devices for pinpointing the delivery of therapeutic compounds into a desired area of the myocardium. As a result, catheter-based techniques for myocardial restoration therapy have evolved from simple intracoronary injections (which are far from perfect, due to the rapid washout of an intravascular compound) to techniques with more efficient therapeutic retention. One of the examples is the TransAccess catheter system with fluoroscopic and intravascular ultrasound guidance, which was used for autologous skeletal myoblast delivery [35]. Currently, the most advanced device for intramyocardial delivery of therapeutic compounds is the NOGA system (Figure 3). The latter allows for the performance of 3D electromechanical mapping of the LV in order to identify target zones and perform precise transendocardial injections of therapeutics. Available data and the authors‘ own experience, derived from large animal models, confirm that the NOGA device is safe and highly effective.

## 5. Conclusions

Less Invasive procedures, coupled with injectable compounds, present a valid platform for a translational restoration protocol, which may be adopted by interventional cardiologists and heart surgeons. Polytherapeutic adjuvants, such as antioxidants, paracrine-active drugs, and anti-inflammatory substances, may be added to the protocols to ensure a sustained myocardial restoration effect.

As discussed in the present paper, among all biomaterials currently used in cardiac tissue engineering, injectable hydrogels, with their potential for minimally invasive delivery and in-vivo breakdown into harmless derivatives, represent the most promising therapeutic option. However, the translational pathway from bench to bedside is challenging and still needs to be explored. It can be anticipated that in the next few decades the role of cell-vehicle compounds in the treatment of ischemic HF patients will expand, and injectable hydrogels will penetrate into the clinical arena to a higher extent.

## Figures and Tables

**Table 1:** Characteristics of the included studies.

Author	Species	Sex/Age	Weight (kg)	Number	Grouping	Aim of the Study
Giordano, C et al., 2013 [19]	Swine, Yorkshire Pig	F/−	8–10	32	Control: 10; CAC: 8; CAC+Matrix: 7; Death: 7	To investigate the effects of biopolymer-supported delivery of circulating angiogenic cells.
Leor, J et al., 2009 [12]	Swine, Domestic	F/-	50–60	58	Death: 22; Exclusion: 1; Control: 16; Intervention: 19	To investigate whether a selective intracoronary injection of alginate solution would result in localised gelation as scaffold in the infarcted tissue.
Matsumura, Y et al., 2019 [13]	Swine, Yorkshire Pig	F/4–5 months	20–30	12	Control: 6; Treatment: 6	To investigate whether the injection of a fully synthetic hydrogel designed for MI treatment was effective in attenuating post-MI LV remodeling.
Qiang Wang et al., 2021 [20]	Chinese Pama Minipig	M/6 months	15–20	45	Control: 15; hUMSC: 15; hUMSC with Collagen: 15	To investigate whether an injectable collagen scaffold promoted the long-term retention of transplanted stem cells.
Yamamoto, T et al., 2001 [21]	Canine, Mongrel	−/Adult	20.3 ± 0.6	28	AGHM-bFGF: 13; bGFG only: 9; AGHM only: 6	To find out if bFGF-impregnated AGHM would enhance collateral development to the infarct area.
Zhou, D et al., 2012 [10]	Canine, Mongrel	−/−	9–14	32	MI: 5; MI + NS: 6; CH: 6; CH + GF: 7; Death: 8	Determine whether TMJR with chitosan scaffolds retained channel patency and enhanced angiogenesis.
Cohen, J.E et al., 2020 [22]	Ovine, Dorset Sheep	M/6–7 months	35–40	21	Saline: 6; HG: 4; NRG: 4; NRG-HG: 7	To evaluate the effectiveness of an NRG-HG therapy to enhance cardiac function.
Contessotto, P et al., 2021 [14]	Ovine, Romanov Sheep	M/8 months	30–40	39	MI only (7 day): 6; MI only (28 day): 8; PBS: 6; ELRs-Hydrogel: 6; Death: 12; Exclusion: 1	To evaluate the effectiveness of an ECM-mimicking hydrogel in modulating post-ischemic.
Li, Y et al., 2021 [23]	Swine, Yucatan mini pigs	M/-	45–50	25	Sham: 3; Saline: 5; agomiR-21-5p: 5; Gel@MSN/miR-NC: 6; Gel@MSN/miR-21-5p: 6	To demonstrate that a microRNA-21-5p delivery system enables both immuno-modification and enhanced angiogenesis for myocardial infarction.
Purcell, B.P et al., 2013 [24]	Swine, Yorkshire Pig	M/−	20	26	Sham:5; MI/Saline: 7; MI/HAMMPS: 7; MI/HAMMPS/rTIMP-3: 7	To investigate whether the localized delivery of an MMP-sensitive biomaterial that releases a recombinant TIMP held promise as a means to interrupt adverse post-MI remodeling.
Chang, M.Y et al., 2016 [25]	Swine, Lanyu Minipigs	M/5 months	22.26 ± 0.78	34	Sham; MI + NS; MI + CB-MNC; MI + HA; MI + CB-MNC/HA	To investigate whether the injection of CB-MNCs combined with hyaluronan hydrogel improved cell therapy efficacy.
Ifkovits, J. L et al., 2010 [15]	Ovine, Dorset Sheep	M/Adult	35–40	21	Control: 9; MeHA high: 7; MeHA low: 5	To compare the effects of two injectable MeHA formulations that exhibit similar degradation but have differential moduli.
Koudstaal, S et al., 2014 [26]	Swine, Dalland Landrace	F/6 months	~70	18	Control (GF): 5; Death: 4; Hydrogel: 4; Hydrogel + GF: 5	To investigate whether the effect of IGF-1/HFG therapy was also effective in the post-MI heart.
Lin, Y.D et al., 2015 [27]	Swine, Minipigs	−/5 months	-	27	5-6 per group: Sham, NS, NF only, MNC only, MNC + NF; Death: 1	To test whether the benefits of an injection of peptide nanofibers continued to persist as the material degraded.
Liu, Y et al., 2006 [11]	Canine, Mongrel	−/−	15–20	18	Control: 6; bFGF alone: 6; bFGF + BDNF: 6	To assess whether the simultaneous application of bFGF- and BDNF-incorporating gelatin hydrogels improved angiogenesis.
Yao Chang, M et al., 2005 [28]	Porcine, Lanyu Minipigs	−/5 months	23.88 (mean)	45	Sham, MI+NS, MI+NF (1 day), MI+MNC (1 day), MI+NF/MNC (1 day), MI+NF/MNC (4 days), MI+NF/MNC (7 days)	To evaluate the therapeutic time window for NF/MNC therapy in acute myocardial infarction.
Rodell, C.B et al., 2016 [16]	Ovine, Dorset	M/Adult	45	22	Saline, GH Hydrogel, DC Hydrogel	To investigate whether soft hydrogels with in-vivo stiffening enhanced therapeutic efficacy to limit LV remodeling and heart failure.
Spaulding, K.A et al., 2020 [17]	Ovine, Dorset Cross-Breed	M, castrated/−	−	14	Control: 7; Treatment: 7	To find out if an injection of a thermoresponsive hydrogel, with ROS scavenging properties, into the MI would decrease ROS.
Takehara, N et al., 2008 [29]	Swine, Yorkshire pigs,	F/-	−	60	Placebo: 15; Gelatin hydrogel: 6; hBMCs: 6; hCDCs: 9; Death: 17; Exclusion: 7	To determine whether the controlled release of bFGF might improve hCDC therapy.
Vu, T.D et al., 2011 [30]	Swine, Yorkshire pig	F/-	65–70	36	Sham: 6; Control: 6; Hydrogel only: 6; PRP-only: 6; Hydrogel + AA: 6; Hydrogel + AA + PRP: 6	To evaluate whether hyaluronic acid-based hydrogel, coupled with PRP, improved host-cell viability.
Traverse, J.H et al., 2019 [18]	First in Human Study	M/F (12/3), 59.6 ± 8.8 years	−	15 patients	Early group (2–12 months post-STEMI): 7; Late group (1–3 years post STEMI): 8	To evaluate the safety and feasibility of transendocardial injections of VentriGel, a cardiac extracellular matrix hydrogel, in early and late post-MI patients with LV dysfunction.

CAC: Circulating Angiogenic Cells; MI: Myocardial Infarction; LV: Left Ventricle; hUMSC: Human Umbilical Mesenchymal Stem Cells; AGHM: Acidic Gelatin Hydrogel Microspheres; bFGF: Basic Fibroblast Growth Factor; NS: Normal Saline; CH: Chitosan Hydrogel; GF: Growth Factor; TMJR: Transmyocardial Jet Revascularization; HG: Hydrogel; NRG: Neuregulin; ELRs: Elastin-like Recombinamers; miR: microRNA; MSN: Mesoporous Silica Nanoparticles; miR-NC: microRNA-Negatice Control; HAMMPS: Matrix Metalloproteinase-Sensitive Hyaluronic Acid Gel; rTIMP-3: Tissue Inhibitors of Metalloproteinase Recombinant Protein; MMP: Matrix Metalloproteinase; CB-MNC: Cord-Blood Mononuclear Cells; HA: Hyaluronic Acid; MeHA: Methacrylated Hyaluronic Acid, NF: Nanofibres; MNC: Mononuclear Cells; BDNF: Brain-Derived Neurotrophic Factor; GH: Guest-Host Hydrogel; ROS: Reactive Oxygen Species; hBMCs: Human Bone Marrow Derived Mesenchymal Cells; hCDCs: Human Cardiosphere-Derived Cells; PRP: Platelet-Rich Plasma; AA: Ascorbic Acid; STEMI: ST-Segment Elevated Myocardial Infarction.

**Table 2:** Summary of outcome assessment—improved cardiac function in treatment groups with hydrogel.

Author	Echo	MRI	CT	PET	Ventricular catheterization	Immunohistochemistry	Masson’s Trichrome Staining	Other
Giordano, C et al., 2013 [19]	LVEF ↑ WMSI ↓			MBF during: Rest ↓, Stress ↑, MFR ↑		BV amount ↑		
Leor, J et al., 2009 [12]	ES area ↓, LV mass ↓					Wall thickness ↑		
Matsumura, Y et al., 2019 [13]		ESV ↓, EDV ~, LVEF ↑, FAC ↑, Scar size ↓, SV ↑				Angiotensin II ↑	Cardiac fibrosis ↓	Stiffness ↑ (Biaxial mechanical)
Qiang Wang et al., 2021 [20]	LVEF ↑ CO ↑, SV ↑, ESV ↓, EDV ↓	LVEF ↑ CO ↑, SV ↑ ESV ↓, EDV ↓	Scar size ↓ Infarct size ↓ LVMV ↓			Cell retention ↑, Arteriole density ↑, Island-/strip- shaped cTnT-positive cells ↑		
Yamamoto, T et al., 2001 [21]					LVEF ~, LVEDP ~, Antegrade flow ↑, Wall motion ~, MBF in ischemic region ↑	BV density ↑		
Zhou, D et al., 2012 [10]						Endothelization ↑, Arteriole and small vessel density ↑, Larger arteriole density ↑	Cardiac fibrosis ~	Size of infarct region ~ (via weighing)
Cohen, J.E et al., 2020 [22]					LVEF ↑, Mean arterial pressure ↑ EDV ↓, ESV ↓, ESPVR ↑			
Contessotto, P et al., 2021 [14]	LVEF ↑					Wall thickness ↑, Collagen fibers ↓ BV density ↑, Cardiomyocyte preservation ↑	Cardiac fibrosis ↓	
Li, Y et al., 2021 [23]	LVEF ↑ EDV ↓, ESV ↓ LV EDd ↑		Scar size ↓ Wall thickness ↑			BV density ↑, BV volume ↑, Infarct size ↓ Immunomodulatory effect ↓	Cardiac fibrosis ↓	
Purcell, B.P et al., 2013 [24]	LVEF ↑, EDV ↓, ESV ↓, Wall thickness ↑, LV mass ↓							Transcriptional activity of myofibroblasts and profibrotic pathways ↓ (mRNA profiling)
Chang, M.Y et al., 2016 [25]	LVEF ↑ IVS thickness ↑				+dp/dt ↑, -dp/dt ↑ LV EDP ↓, EDV ↓	Cell retention ↑ BV density ↑ EC differentiation ↑		Scar size ↓ Wall thickness ↑ (Via gross cross-section)
Ifkovits, J. L et al., 2010 [15]	LVEF ↑, ESV ↑, EDV ↑, CO ↑					Wall thickness ↑ Infarct area ↓		
Koudstaal, S et al., 2014 [26]	LVEF ↑ EDV ↑, ESV ↑, FAS ↑, PRSW ↑					Cardiomyocyte hypertrophy ↓, Cardiomyocyte proliferation ↑, Fibrosis extent ↓, BV density ↑, C-kit number ↑		
Lin, Y.D et al., 2015 [27]	LVEF ↑, PSV ↑, E/A ratio ↓				SV ↑, AE ↑, +dP/dt ↑, -dP/dt ↑, PRSW ↑, Emax ↑, T ↓, ESV ↓, EDV ↓, ESP ↑, EDP ↓	BV density ↑		
Liu, Y et al., 2006 [11]		LVEF ↑			MBF ↑	BV density ↑, bFGF expression ↑ BDNF expression ↑, Distribution of bFGF and BDNF positive cells ~		
Yao Chang, M et al., 2005 [28]	LVEF ↑				IVS thickness ↑ Systolic function ↑ EDP ↑, EDV ↑, Emax ↑	Infarct size ↓, Infarct length ratio ↓ BV density ↑, Blood flow ↑	Cardiac fibrosis ↓	Stem cell retention ↑ (Confocal microscopy with Dil and DAPI staining)
Rodell, C.B et al., 2016 [16]		LVEF ↑ EDV, ESV ↓ LV wall thickness ↑						Myofiber stress reduction ↑ (Via FE simulation model, dimensions of model obtained via MRI)
Spaulding, K.A et al., 2020 [17]	LVEF ↑ (2 wk), LVEF ↓ (6 wk), EDV ↑ ESV ↑	LV wall thickness ↑ Demembranated muscle force ↑				Levels of ROS in BZ ↓ FL-MMP-2 ↓		SV ↑ (2 weeks) SV ↓ (6 weeks) PCWP ↑ (Via Swan Ganz)
Takehara, N et al., 2008 [29]	LVEF ↑, RWM ↑ Myocardial perfusion ↑ Infarct size ↓	Stem cell retention ↑				Myocyte conversion ↑		
Vu, T.D et al., 2011 [30]	LVEF ↑ FAC ↑ EDV ↓	LV mass ↓ LV collagen area fraction ↓ Scar size ↓				BV density ↑ BV amount ↑		
Traverse, J.H et al., 2019 [18]		LVEF ~, EDV ↓, ESV ↓ Scar size ~, Viable mass ↑						BNP ↓, 6-min walk test distance ↑ NYHA class ↑, MLWHFQ score ↑

↑ Represents a higher result for the parameter in the treatment group, as compared to the control group. ↓ Represents a lower result for the parameter in the treatment group, as compared to the control group. LVEF: Left Ventricular Ejection Fraction; WMSI: Wall Motion Score Index; MBF: Myocardial Blood Flow; MFR: Myocardial Flow Reserve; BV: Blood Vessel; ES: End-Systolic; LV: Left Ventricle; ESV: End-Systolic Volume; EDV: End-Diastolic Volume; FAC: Fractional Area Change; SV: Stroke Volume; CO: Cardiac Output; LVMV: Left Ventricle Mass Volume; cTnT: Cardiac Troponin-T; LVEDP: Left Ventricle End-Diastolic Pressure; ESPVR: End-Systolic Pressure-Volume Relationship; LVEDd: Left Ventricular End-Diastolic Dimension; IVS: Interventricular Septum; +dp/dt: Measure of Systolic Function; -dp/dt: Measure of Diastolic Function; FAS: Fractional Area Shortening; PRSW: Preload Recruitable Stroke Work; PSV: Peak of Systolic Velocity; E/A: E-wave to A-wave Ratio; AE: Arterial Elastance; Emax: Maximum Chamber Elasticity; T: Time constant of Left Ventricular Pressure Decay; ESP: End-Systolic Pressure; EDP: End-Diastolic Pressure; bFGF: Basic Fibroblast Growth Factor; BDNF: Brain-Derived Neurotrophic Factor; DAPI: 4’,6-diamidino-2-phenylindole; ROS: Reactive Oxygen Species; BZ: Border Zone; FL-MMP-2: Full Length Matrix Metalloproteinase 2; PCWP: Pulmonary Artery Wedge Pressure; RWM: Regional Wall Motion; BNP: B-Type Natriuretic Peptide; NYHA: New York Heart Association; MLWHFQ: Minnesota Living with Heart Failure Questionnaire.

**Table 3:** Hydrogel characterization and mode of delivery.

Author	Method of MI Creation	Artery Involved	Cell Delivered	Type of Matrix	Method of Delivery to Myocardium
Giordano, C et al., 2013 [19]	Left thoracotomy with ameroid constrictor	Proximal LCx	CAC	Type-I rat tail collagen cross-linked with glutaraldehyde (BD Bioscience, Oakville, Canada)	Open; Intramyocardial
Leor, J et al., 2009 [12]	Balloon occlusion	Mid-LAD artery	Acellular	Sodium alginate (VLVG, NovaMatrix, FMC Biopolymers, Drammen, Norway)	Intracoronary
Matsumura, Y et al., 2019 [13]	Left thoracotomy with suture ligation	Between 1st and 2nd diagonal branches	Acellular	Synthetic Hydrogel: Poly (NIPAAm-co-HEMA-co-MAPLA) (Sigma-Aldrich, USA)	Open; Intramyocardial
Qiang Wang et al., 2021 [20]	Left thoracotomy with suture ligation	LAD distal to origin of 2nd branch	hUMSC	Bovine collagen	Open; Intramyocardial
Yamamoto, T et al., 2001 [21]	Left thoracotomy with suture ligation	LAD between 1st and 2nd diagonal branches	bFGF	AGHM	Open; Subepicardial implantation
Zhou, D et al., 2012 [10]	Left thoracotomy with suture ligation	LAD below 1st diagonal branch	VEGF165	Temperature-responsive Chitosan hydrogel	Open; Transmyocardial jet revascularization
Cohen, J.E et al., 2020 [22]	Left thoracotomy with suture ligation	2nd and 3rd diagonal branches of LAD	NRG (R&D Systems, Minneapolis, MN, USA)	HEMA-HA based hydrogel (Lifecore Biomedical Inc., Chaska, MN, USA)	Open; intramyocardial
Contessotto, P et al., 2021 [14]	Left thoracotomy with suture ligation	LAD from 1st diagonal branch, moving distally till apex	Acellular	ELRs hydrogel	Open; Intramyocardial
Li, Y et al., 2021 [23]	Left thoracotomy with suture ligation	1st two obtuse marginal arteries of LCx	MSN/miR-21-5p complex	Injectable hydrogel matrix	Open; Intramyocardial
Purcell, B.P et al., 2013 [24]	Left thoracotomy with suture ligation	1st two obtuse marginal arteries of LCx	Full-length rTIMP-3	Hyaluronic acid-based hydrogel with MMP	Open; Intramyocardial
Chang, M.Y et al., 2016 [25]	Left thoracotomy with suture ligation	Mid-LAD	CB-MNC	Hyaluronic acid hydrogel	Open; Intramyocardial
Ifkovits, J. L et al., 2010 [15]	Left thoracotomy with suture ligation	LAD and 2nd diagonal coronary artery	Acellular	Methacrylated hyaluronic acid macromers (MeHA) hydrogel	Open; Intramyocardial
Koudstaal, S et al., 2014 [26]	75 min intracoronary balloon occlusion	LCx	IGF-1/HGF	UPy hydrogel	Open; Intramyocardial
Lin, Y.D et al., 2015 [27]	Left thoracotomy with suture ligation	Mid-LAD	MNCs	Peptide nanofibers	Open; Intramyocardial
Liu, Y et al., 2006 [11]	Left thoracotomy with suture ligation	LAD distal to 1st diagonal branch	bFGF, BDNF	Gelatin hydrogel (Boster Bioengineering Company, Wuhan, China)	Open; Intramyocardial
Yao Chang, M et al., 2005 [28]	Left thoracotomy with suture ligation	Mid-LAD	Bone marrow MNC	Peptide nanofibers	Open; Intramyocardial
Rodell, C.B et al., 2016 [16]	Left thoracotomy with suture ligation	Selective ligation of obtuse marginal branches	Acellular	Guest-host hydrogels; Dual-crosslinking hydrogels	Open; Intramyocardial
Spaulding, K.A et al., 2020 [17]	Left thoracotomy with suture ligation	LAD and its diagonal branches	Acellular	NIPAAm-PEG1500 hydrogel (Sigma-Aldrich, USA)	Open; Intramyocardial
Takehara, N et al., 2008 [29]	90 min intracoronary balloon occlusion, followed by reperfusion	LAD	bFGF (Kaken Pharmaceutical Co., Tokyo, Japan)	Gelatin hydrogel	Open; Intramyocardial
Vu, T.D et al., 2011 [30]	Left thoracotomy with suture ligation	Proximal LCx	Platelet-rich plasma	Hyaluronate Gelatin (Glycosan BioSystems Inc, Salt Lake City, UT, USA)	Open; Intramyocardial
Traverse, J.H et al., 2019 [18]	Patients with 1st STEMI treated by PCI within past 60 days to 3 years with moderate LV dysfunction.	−	Acellular	VentriGelTM—ECM from decellularized porcine myocardium	Transcatheter delivery through endocardium into myocardium

LCx: Left Cirumflex Artery; CAC: Circulating Angiogenic Cells; LAD: Left Anterior Descending Artery; hUMSC: Human Umbilibal Mesenchymal Stem Cell; bFGF: Basic Fibroblast Growth Factor; AGHM: Acidic Gelatin Hydrogel Microspheres; VEGF: Vascular Endothelial Growth Factor; NRG: Neuregulin; HEMA-HA: Hyaluronic Acid Macromers with Hydroxyethyl Methacrylate Group Modification; ELRs: Elastin-like Recombinamers; MSN: Mesoporous Silica Nanoparticles; miR: microRNA; rTIMP-3: Tissue Inhibitors of Metalloproteinase Recombinant Protein; MMP: Matrix Metalloproteinase; CB-MNC: Cord Blood Mononuclear Cells; MeHA: Methacrylated Hyaluronic Acid; IGF-1: Insulin-like Growth Factor 1; HGF: Hepatocyte Growth Factor; UPy: Ureidopyrimidinone; MNCs: Mononuclear Cells; BDNF: Brain-Derived Neurotrophic Factor; NIPAAm: N-Isopropyl Acrylamide; PEG: Polyethylene Glycol.

## Data Availability

Not applicable.

## Supplementary Materials

The following are available online at https://www.mdpi.com/article/10.3390/biomedicines9060595/s1, Figure S1 Full Search Strategy and Figure S2 PRISMA flow diagram.

## Abbreviations

AA	Ascorbic Acid
AE	Arterial Elastance
AGHM	Acidic Gelatin Hydrogel Microspheres
BDNF	Brain-Derived Neurotrophic Factor
BNP	B-type Natriuretic Peptide
bFGF	Basic Fibroblast Growth Factor
BNDF	Brain-Derived Neurotrophic Factor
BV	Blood Vessel
BZ	Border Zone
CACs	Circulating Angiogenic Cells
CB-MNC	Cord Blood Mononuclear Cells
CH	Chitosan Hydrogel
CI	Cardiac Index = SV x (heart rate)/BSA/10
CO	Cardiac Output
cTnT	Cardiac Troponin-T
DAPI	4’,6-diamidino-2-phenylindole
DC	Dual-Crosslinking Hydrogel
+Dp/Dt	Measure of Systolic Function
-Dp/Dt	Measure of Diastolic Function
E/A	E-wave to A-wave Ratio
EC	Endothelial Cell
ECM	Extracellular Matrix
EDV	End-Diastolic Volume
EDP	End Diastolic Pressure
ELRs	Elastin-Like Recombinamers
Emax	Maximum Chamber Elasticity
ES	End-Systolic
ESP	End-Systolic Pressure
ESPVR	End-Systolic Pressure-Volume Relationship
ESV	End-Systolic Volume
FAC	Fractional Area Change
FAS	Fractional Area Shortening
FL-MMP-2	Full Length Matrix Metalloproteinase 2
GF	Growth Factor
GH	Guest-Host Hydrogel
HA	Hyaluronic Acid
HAMMPS	Matrix Metalloproteinase-Sensitive Hyaluronic Acid Gel
hBMCs	Human Bone Marrow Derived Mesenchymal Cells
hCDCs	Human Cardiosphere-Derived Cells
HEMA	Hydroyethyl (Methacrylate)
HEMA-HA	Hyaluronic Acid Macromers with HEMA Group Modification
HF	Heart Failure
HGF	Hepatocyte Growth Factor
HG	Hydrogel
hUMSC	Human Umbilical Mesenchymal Stem Cells
IGF-1	Insulin-Like Growth Factor 1
IVS	Interventricular Septum
LAD	Left Anterior Descending Artery
LCx	Left Circumflex Artery
LV	Left Ventricle
LV EDd	Left Ventricular End-Diastolic Dimension
LVEDP	Left Ventricular End Diastolic Pressure
LV ESV	Left Ventricular End-Systolic Volume
LVEF	Left Ventricular Ejection Fraction
LVMV	Left Ventricle Mass Volume
MAPLA	Methacrylate Polylactide
MBF	Myocardial Blood Flow
MeHA	Methacrylated Hyaluronic Acid
MFR	Myocardial Flow Reserve
MI	Myocardial Infarction
miR	microRNA
miR-NC	microRNA-Negative Control
MLWHFQ	Minnesota Living With Heart Failure Questionnaire
MMP	Matrix Metalloproteinase
MNC	Mononuclear Cells
MSN	Mesoporous Silica Nanoparticles
NF	Nanofibres
NIPAAm	N-Isopropyl Acrylamide
NRG	Neuregulin
NS	Normal Saline
NRG	Neuregulin
NYHA	New York Heart Association
PBS	Phosphate-Buffered Saline
PCI	Percutaneous Coronary Intervention
PCWP	Pulmonary Artery Wedge Pressure
PEG	Polyethylene Glycol
PRP	Platelet-Rich Plasma
PRSW	Preload Recruitable Stroke Work
PSV	Peak of Systolic Velocity
ROS	Reactive Oxygen Species
RWM	Regional Wall Motion
rTIMP-3	Tissue Inhibitors of Metalloproteinase Recombinant Protein
STEMI	ST-Segment Elevated Myocardial Infraction
SV	Stroke Volume
T	Time constant of Left Ventricular Pressure Decay
TMJR	Transmyocardial Jet Revascularization
UPy	Ureidopyrimidinone
VEGF	Vascular Endothelial Growth Factor
WMSI	Wall Motion Score Index
