# Supplementary material for: Targeted Myocardial Restoration with Injectable Hydrogels—In Search of The Holy Grail in Regenerating Damaged Heart Tissue
Source: Biomedicines. 2021 May 24;9(6):595. doi: 10.3390/biomedicines9060595 (PMC8225139; doi:10.3390/biomedicines9060595)
Supplement: Supplementary file 1 [file biomedicines-09-00595-s001.zip › biomedicines-1211116 Figure S1.pdf]

Supplementary Figure S1: Full Search Strategy

Keywords used in database literature searches

Concept 1: Injectable Hydrogels

1) Hydrogels [**A1**]
2) Extracellular matrix hydrogels [**B1**]
3) Tissue engineering [**C1**]

Concept 2:

1) Myocardial infarction [**A2**]

Concept 3: Myocardial restoration therapy

1) Myocardial infarction therapy [**A3**]
2) Cardiac stem cell therapy [**B3**]
3) Cell-based therapy [**C3**]

Table S1: Number of Results Retrieved with Each Permutation of Key Words

Permutation	Key Word	Number of search results		
		PubMed	Embase	Web of Science
**A1A2**	Hydrogels AND Myocardial infarction	453	277	25
**A1A3**	Hydrogels AND Myocardial Infarction Therapy	370	158	1
**A1B3**	Hydrogels AND Cardiac stem cell therapy	206	134	2
**A1C3**	Hydrogels AND Cell-based therapy	455	197	10
**B1A2**	Extracellular matrix hydrogels AND Myocardial infarction	78	49	0
**B1A3**	Extracellular matrix hydrogels AND Myocardial infarction therapy	62	29	0
**B1B3**	Extracellular matrix hydrogels AND Cardiac stem cell therapy	45	37	0
**B1C3**	Extracellular matrix hydrogels AND Cell-based therapy	88	51	0
**C1A2**	Tissue engineering AND Myocardial infarction	1373	3189	25
**C1A3**	Tissue engineering AND Myocardial infarction therapy	933	1732	1
**C1B3**	Tissue engineering AND Cardiac stem cell therapy	1081	1998	6
**C1C3**	Tissue engineering AND Cell-based therapy	2742	3139	32
**A2B3**	Myocardial infarction AND Cardiac stem cell therapy	3324	4888	39
**A2C3**	Myocardial infarction AND Cell-based therapy	619	838	18
**Total Number of Results Per Database**		11829	16716	159
**Total Number of Results**		28704		
