# Supplementary material for: Targeted Myocardial Restoration with Injectable Hydrogels—In Search of The Holy Grail in Regenerating Damaged Heart Tissue
Source: Biomedicines. 2021 May 24;9(6):595. doi: 10.3390/biomedicines9060595 (PMC8225139; doi:10.3390/biomedicines9060595)
Supplement: Supplementary file 1 [file biomedicines-09-00595-s001.zip › biomedicines-1211116 Figure S2.pdf]

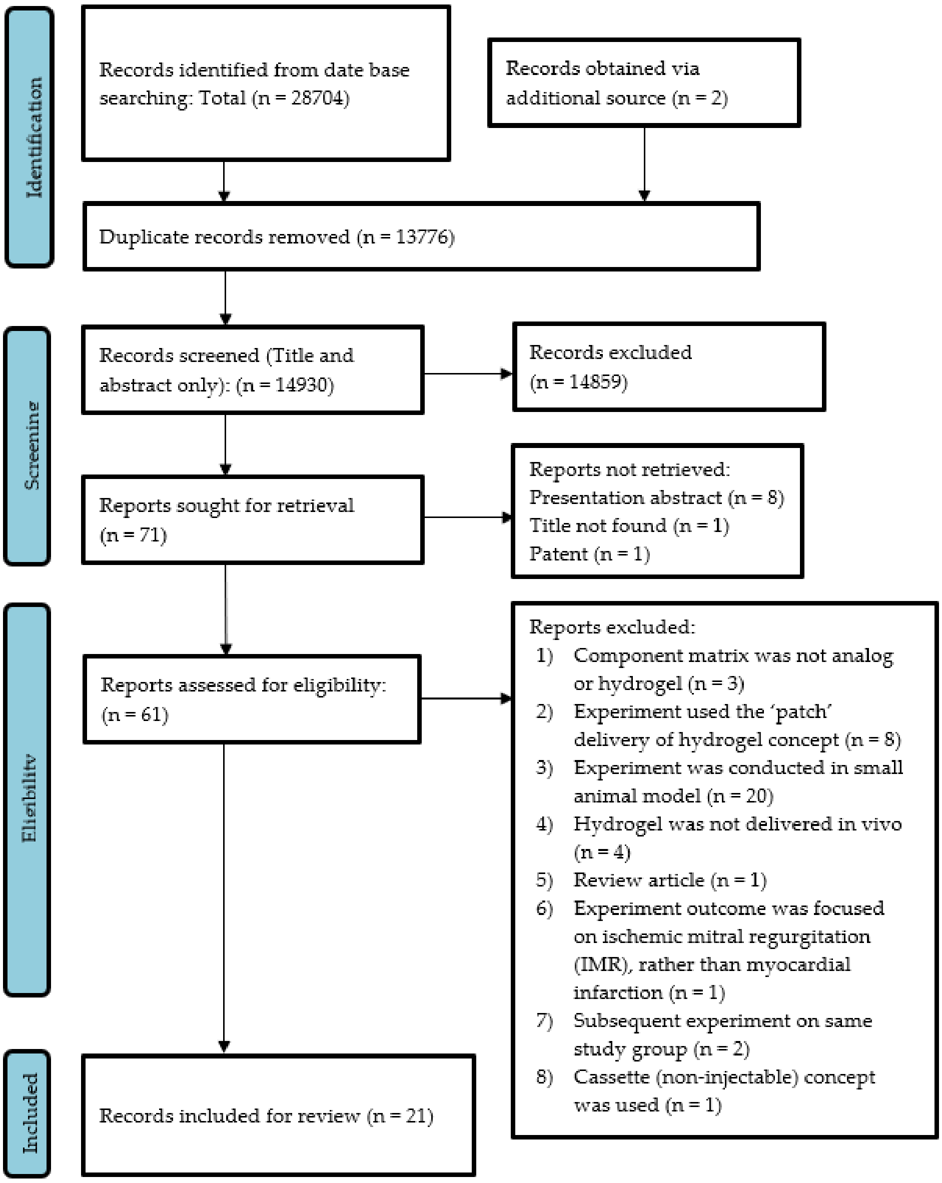

Figure S2. PRISMA chart illustrating our process of obtaining the 21 included articles. Our initial search produced 28704 studies and 2 study was retrieved from alternative sources. Out of this initial pool of studies, 13776 remained after duplicates were removed. With 71 irrelevant records excluded based on their titles and abstracts, we reviewed the full texts of 61 articles, of which 40 were excluded and 21 remained for inclusion in our study.
